# Using a tablet to understand the spatial and temporal characteristics of complex upper limb movements in chronic stroke

**DOI:** 10.1371/journal.pone.0311773

**Published:** 2024-11-18

**Authors:** Devin Sean Austin, Makenna J. Dixon, Joanna E. Hoh, Duncan Thibodeau Tulimieri, Joshua G. A. Cashaback, Jennifer A. Semrau

**Affiliations:** 1 Graduate Program in Biomechanics and Movement Science (BIOMS), University of Delaware, Newark, Delaware, United States of America; 2 Department of Kinesiology and Applied Physiology, University of Delaware, Newark, Delaware, United States of America; 3 Department of Biomedical Engineering, University of Delaware, Newark, Delaware, United States of America; Aalto University School of Science and Technology: Aalto-yliopisto Insinooritieteiden korkeakoulu, FINLAND

## Abstract

Robotic devices are commonly used to quantify sensorimotor function of the upper limb after stroke; however, the availability and cost of such devices make it difficult to facilitate implementation in clinical environments. Tablets (e.g. iPad) can be used as devices to facilitate rehabilitation but are rarely used as assessment tools for the upper limb. The current study aimed to implement a tablet-based Maze Navigation Task to examine complex upper-limb movement in individuals with chronic stroke. We define complex upper-limb movement as reaching movements that require multi-joint coordination in a dynamic environment. We predicted that individuals with stroke would have more significant spatial errors, longer movement times, and slower speeds compared to controls with increasing task complexity. Twenty individuals with chronic stroke who had a variety of arm and hand function (Upper extremity Fugl-Myer 52.8 ± 18.3) and twenty controls navigated eight pseudorandomized mazes on an iPad using a digitizing stylus. The task was designed to elicit reaching movements engaging both the shoulder and elbow joints. Each maze became increasingly complex by increasing the number of 90° turns. We instructed participants to navigate each maze as quickly and accurately as possible while avoiding the maze’s boundaries. Sensorimotor behavior was quantified using the following metrics: Error Time (time spent hitting or outside boundaries), Peak Speed, Average Speed, and Movement Time, Number of Speed Peaks. We found that individuals with stroke had significantly greater Error Time for all maze levels (all, p < 0.01), while both speed metrics, Movement Time and Number of Speed Peaks were significantly lower for several levels (all, p < 0.05). As maze complexity increased, the performance of individuals with stroke worsened only for Error Time while control performance remained consistent (p < 0.001). Our results indicate that a complex movement task on a tablet can capture temporal and spatial impairments in individuals with stroke, as well as how task complexity impacts movement quality. This work demonstrates that a tablet is a suitable tool for the assessment of complex movement after stroke and can serve to inform rehabilitation after stroke.

## Introduction

Sensorimotor impairments of the upper limb affect approximately 70% of individuals with stroke [1–3]. These impairments include diminished motor function, proprioceptive dysfunction, and overall impairments in limb coordination [4–8]. Typically, these impairments are evaluated using clinical assessments to determine the effectiveness of clinical rehabilitation and to develop targeted rehabilitation interventions; however, current clinical assessments often lack precision, sensitivity, and can be time-consuming to administer in a clinic [9–14]. These barriers to precise assessment of upper limb motor function make it difficult to capture accurate measures of sensorimotor function after stroke and to understand more nuanced behavior–such as impairments in the timing of movements and directional planning.

Several groups have highlighted the advantages of robotic devices over the past two decades as an objective assessment of upper limb function after stroke [5, 15–17].

These devices can quantify behavior using continuous rather than ordinal scales, have high sensitivity, require minimal training to use, and provide rapid assessments of upper limb impairments based on the kinematics of reaching. For example, Coderre et al. demonstrated the effectiveness of the Kinarm Robotic Exoskeleton to assess upper limb function in individuals with stroke when compared to common clinical assessments of upper limb impairments [18]. The authors had participants perform an eight-target visually guided reaching task while examining several sensorimotor characteristics derived from reaching movements. Examining these characteristics enabled the authors to reliably identify impairments in individuals with stroke with similar accuracy as the Chedoke McMaster Stroke Assessment Scale (a common upper limb clinical assessment), demonstrating the effectiveness of robotic devices in upper limb assessments. Similar results were found by Colombo et al. who developed and demonstrated the effectiveness of two robotic devices: a one-degree-of-freedom robot for wrist rehabilitation and a two-degree-of-freedom robot for elbow-shoulder rehabilitation. Using these devices, the authors systematically assessed upper limb impairments at various stages of rehabilitation in individuals with chronic stroke. The authors established a positive relationship between the comprehensive outcomes provided by each device and the Fugl-Meyer scores of individuals with chronic stroke. This relationship was both statistically significant and moderately strong; which denotes the effectiveness of robotic devices for monitoring the progression of rehabilitation [19].

Recent work has demonstrated that examining limb kinematics can significantly improve rehabilitation programs, suggesting a critical need for devices that can better inform and guide clinical practice [20–22]. While robotics can uniquely quantify limb kinematics, these devices are often inaccessible, cost between $10,000 to $300,000 USD, and are predominantly found in university-based rehabilitation centers [23]. These characteristics make such assessment tools considerably out of reach for rural and financially disadvantaged centers, further widening the accessibility gap in stroke rehabilitation [24]. Here, there is considerable room for the development of more accessible technology (e.g. tablet devices) for the assessment of sensorimotor impairments in individuals with stroke.

Tablet devices have seen extensive use for rehabilitation in aphasia and monitoring of rehabilitation statuses [25–29]. Cock et al. demonstrated the feasibility of their Speech Therapy App using a tablet for administering aphasia therapy in individuals with acute stroke. Utilizing their application, individuals with aphasia practiced linguistic skills for 30 minutes per day without the assistance of a clinician. The authors found that participants enjoyed using the application and significantly benefited from the additional practice by demonstrating improved recovery [25]. Additionally, Kurland et al. developed an application to monitor aphasia rehabilitation during 6-months of independent home care. The authors were able to capture substantial improvements throughout the 6-month care period, demonstrating the effectiveness of unsupervised home therapy using a tablet during aphasia rehabilitation [27]. Surprisingly, these devices have seen limited use for the evaluation or assessment of upper limb sensorimotor function in stroke [30–32]. Recent studies have examined several aspects of motor function; however, their conclusions are limited to singular kinematic results such as reaction time or hand dexterity [29, 33]. Additionally, these studies primarily examine simple, point-to-point movements involving a limited range of motion in a singular plane. However, these types of movements often lack generalizability to real world behaviors that involve multi-joint coordination and navigation around obstacles. Thus, there is a critical need for the development of rapid, objective, and easy-to-administer assessment tools for the upper limb that examine complex reaching movements that can also generalize to real-world behaviors.

To address this need, the current study aimed to design and implement a tablet-based Maze Navigation Task to examine upper limb sensorimotor behavior in individuals with chronic stroke during complex reaching movements. Our novel design takes ~10 minutes to administer and requires users to engage in multi-joint movements to complete the task. Through examination of upper limb behavior with our design, we objectively evaluate several sensorimotor characteristics of complex upper limb movements representing speed, time, and accuracy. Given prior studies have demonstrated impaired upper limb function in individuals with chronic stroke, we expected our novel task to effectively capture these deficits [29, 31, 34]. We predicted that individuals with chronic stroke will show slower speeds, increased errors, increased corrective movements, and take longer to complete the task compared to controls. We also predicted that as maze complexity increases, the performance of the individuals with stroke will significantly worsen in both temporal and spatial measures when compared to controls.

## Methods

### Participant information

Twenty controls and twenty individuals with stroke participated in this cross-sectional study. Control participants were recruited from the immediate regions surrounding the University of Delaware while individuals with stroke were recruited through the University of Delaware Stroke Research Registry. All participants completed the tablet-based Maze Navigation Task (described below) in a single study visit lasting less than one hour. The following exclusion criteria were applied to all participants: history of a significant upper-body injury (e.g., shoulder replacement), neurological impairment other than stroke (e.g., Parkinson’s Disease), or disease that may impact limb sensation (e.g., Diabetic Neuropathy). Control participants had no previous history of stroke or other neurological injuries. Inclusion criteria specific to the individuals with stroke included single-occurrence stroke, stroke in the chronic phase (>6 months post-stroke), no presence of a visual field cut, no presence of visuospatial neglect, and a Montreal Cognitive Assessment (MOCA) > 18 [35]. No individuals with stroke were excluded from this study based on upper limb impairment level due to the need to explore the feasibility of our approach with across different levels of impaired motor function which is representative of the population of individuals with stroke at large.

We must note that one individual with stroke was recruited with a MOCA of 16. However, this person had clinically-confirmed expressive aphasia and provided confirmation for understanding task instructions. This study was approved by the University of Delaware Institutional Review Board, and all participants provided written informed consent.

### Experimental setup

**Fig 1:**
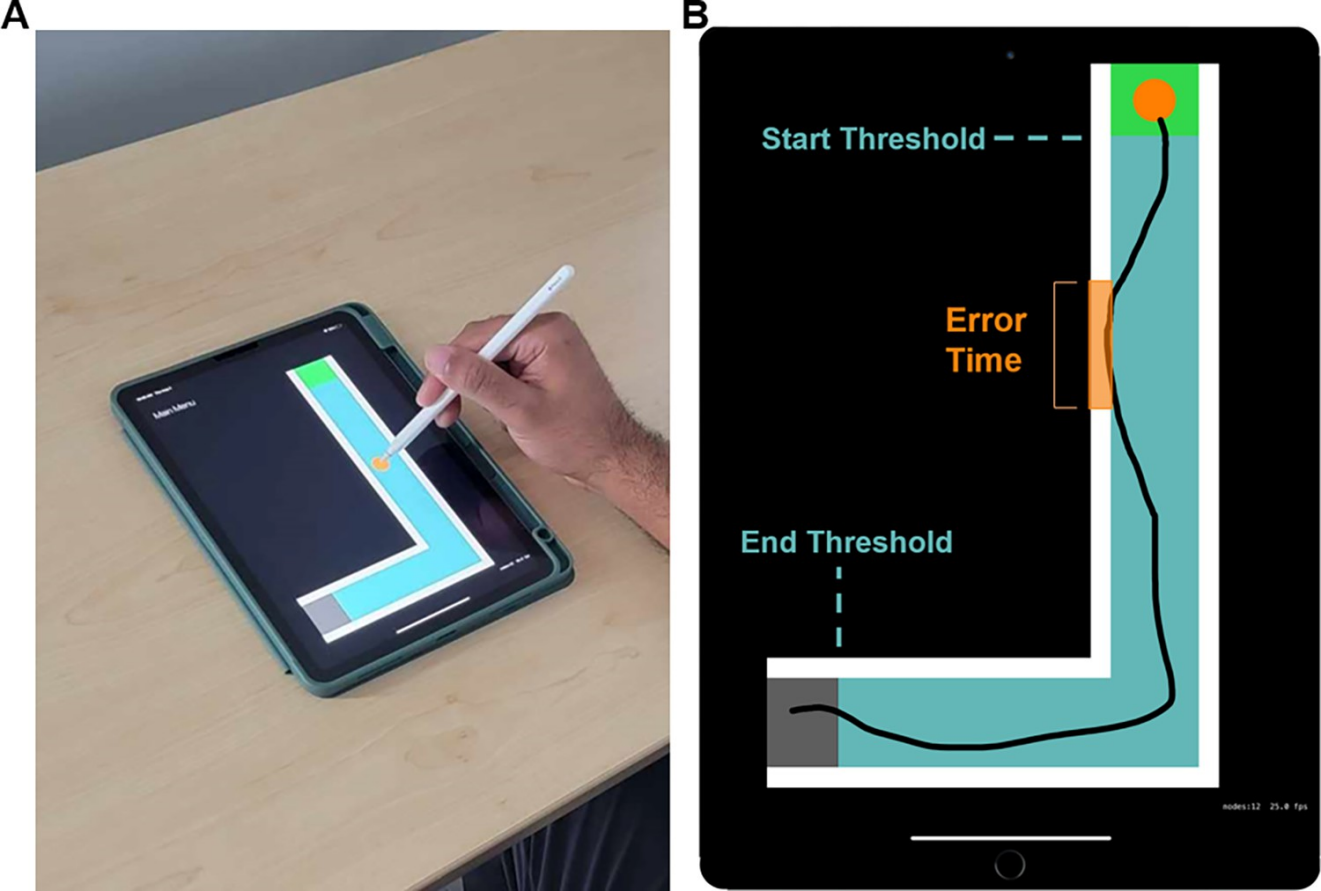
(A) Experimental setup depicting the Apple iPad Pro (10^th^ generation) and Apple 2^nd^ generation digitizing pen. (B) Overhead view of the tablet screen with example tracing movement for Level 1 of the Maze Navigation Task. Using an Apple pencil, participants drag the orange cursor through the maze while avoiding the white walls. Movement is recorded between the start and end positional thresholds noted by the borders of the green and grey regions, respectively. The time the orange cursor is in contact with or goes outside the white maze boundary is recorded and noted as Error Time.

Kinematic data was recorded using an Apple iPad Pro (10^th^ generation) and Apple 2^nd^ generation digitizing pen (**Fig 1A**). When completing the task on the iPad; the participant sat comfortably at a desk holding a digitizing pen. The iPad was placed perpendicular to the participant at approximately 5 cm from the edge of the desk. Participants could move the iPad to a comfortable position at their discretion; however, we requested that they did not move the device more than 2 cm in any direction. Assistive devices for grasping the digitizing pen were offered to individuals with stroke who had issues with grip strength and/or limitations in hand dexterity. These devices include a 7 cm long cylindrical foam (2 cm in diameter and 0.5 cm thick) to reduce the grip strength required to hold the pen, an adaptive universal cuff that gave participants the option to hold the digitizing pen without using their fingers, and a strip of Dycem non-stick material that was wrapped around the pen to reduce the chance of the pen slipping out of the participant’s hand. One individual with stroke used both a foam tube and Dycem (FMA Score = 19) and two individuals (FMA Scores = 11) used all three adaptive devices due to trouble maintaining their grip. The two individuals using all three devices also received assistance from an occupational therapist to initially place their hand–which is holding the pen–on the device; and reset their position between trials when necessary. Similar methods have been previously described from our group [34].

### Maze Navigation Task

Using the iPad, participants completed a Maze Navigation Task to navigate eight pseudorandomly generated mazes (**Fig 1**). Each maze was generated from a custom maze generation algorithm programmed based on a two-dimensional integer lattice random walk in MATLAB R2021a and the iPad application used for the experiment was created in XCode 12.5 [36]. The maze generation algorithm itself generated eight, single-path, mazes based on three parameters needed to create a simple maze: path length, path width, and the specified number of turns. These are the minimum requirements needed to generate several unique mazes. During the creation of the mazes, the path length was held constant at ten units with a width of one unit, which was scaled when implemented on the iPad to a new path length of 21.07 ± 1.16 cm long and a path width of 1.93 ± 0.73 cm wide. The resulting size of each maze was chosen to minimize the chance of only using wrist movements to navigate the maze. If a generated maze collided with itself due to the increasing number of turns, then that maze was re-generated.

**Fig 2:**
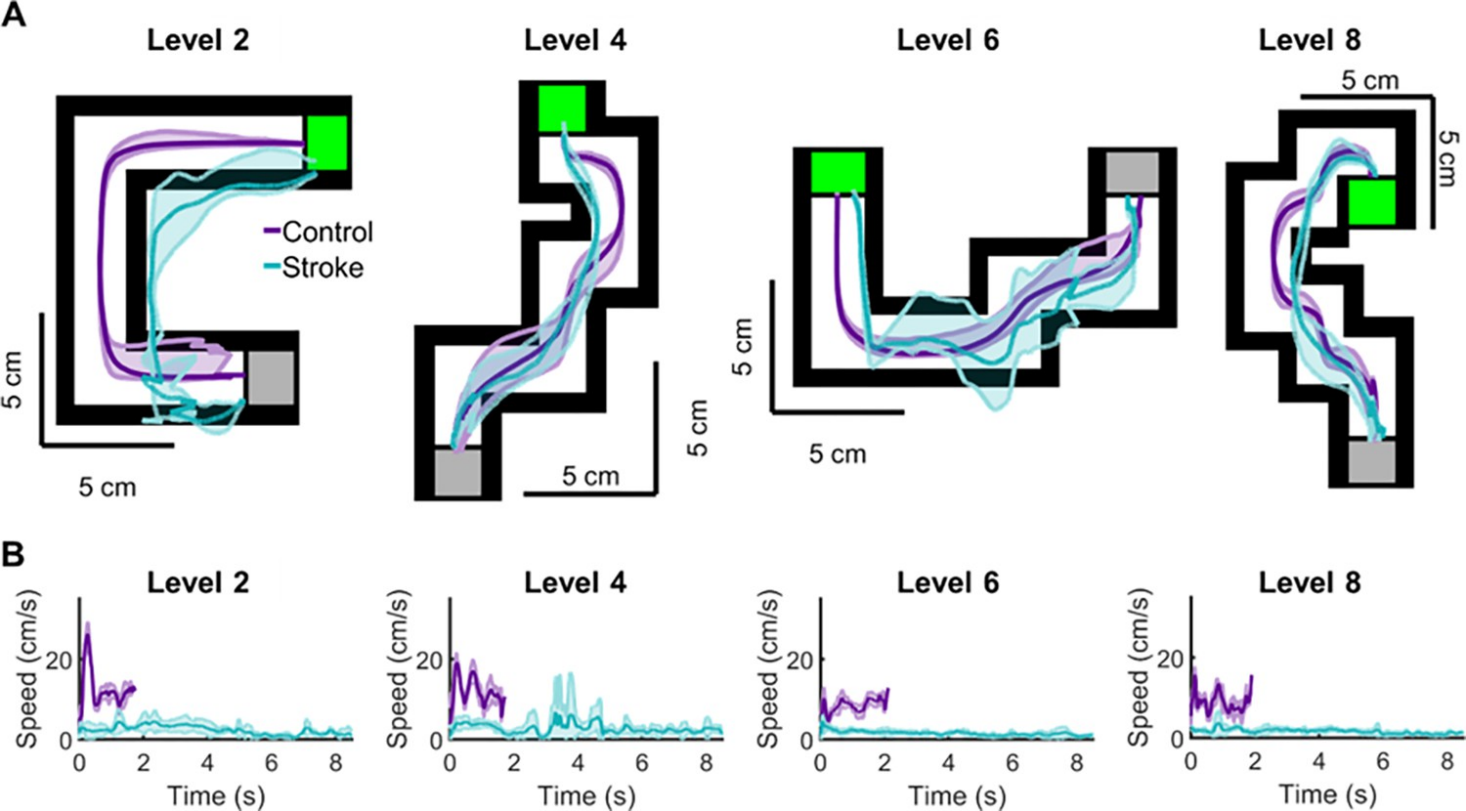
Exemplar kinematic hand traces on the Maze Navigation Task for Levels 2, 4, 6, and 8. Data is averaged across five trials, with the shaded region indicating standard deviation for hand position (A) and hand speed (B) for an exemplar control (purple) and an individual with stroke (cyan). A) Hand position as a function of level performance shows that the individual with stroke made more errors and had more difficulty maintaining their hand path within the borders of the maze. B) Hand speed as a function of level performance shows that the individual with stroke took consistently longer and had overall reduced hand speed compared to the control participant.

Participants were instructed to drag the cursor through the maze as quickly and accurately as possible while avoiding the walls surrounding the maze. All participants completed the task twice–once with each hand. Each participant’s starting hand was counterbalanced to control for practice effects. Participants were required to drag an orange cursor from a starting region, noted by a green box placed at the beginning of the maze, to a finish region, noted by a gray box placed at the end of the maze, as quickly and accurately as possible while avoiding the maze’s boundary. The boundary was defined by white walls that encapsulated the maze (**Fig 1)**. If contact was made with a boundary or the participant left the maze, the white walls would turn purple to notify participants of their error. Participants completed five trials for each maze level, with each maze becoming more complex by increasing the number of 90° turns (e.g. Level 1 = 1 turn, Level 8 = 8 turns). Participants started the experiment by performing three practice trials on a single maze with no turns, then advanced through five trials for each level sequentially. Level 1 is pictured in **Fig 1B** and even levels are pictured in **Fig 2A.**

### Clinical evaluation procedure

All individuals with stroke underwent a series of clinical assessments by one of two study clinicians, a Physical Therapist or an Occupational Therapist with expertise in stroke. Assessments included: the Fugl-Meyer Assessment (FMA) to measure arm and hand motor function, the Function Independence Measure (FIM) to measure functional ability, Thumb Localizer Test (TLT) to measure position sense, Behavioral Inattention Test (BIT) to assess for visuospatial neglect, Montreal Cognitive Assessment (MoCA) to assess cognitive function, Purdue Pegboard (PPB) to assess manual dexterity, and the Edinburgh Handedness Scale to determine handedness [11, 37–42].

### Kinematic data analysis

Several kinematic measures were calculated to quantify the ability to execute complex movements. Measures were calculated for each trial, then averaged within an individual for that parameter, across trials, within each level. The green starting region’s border facing the maze determined the start threshold (**Fig 1B**). Similarly, the end threshold used the grey finish region. Only hand position and speed between start and end thresholds were used when calculating the following kinematic measures:

1. Peak Speed (cm/s)—the fastest speed achieved between start and end movement through the maze
2. Average Speed (cm/s)—average hand speed during the movement.
3. Movement Time (s)—the amount of time it took participants to complete the maze.
4. Error Time (%)—the percentage of time participants spent in contact or outside a maze boundary relative to their Movement Time.5. Number of Speed Peaks (arbitrary units)–the number of corrective movements made during a reach derived from hand speed.

### Statistical analysis

Our initial analysis determined whether there were differences in performance based on handedness within the control group. Here, we used paired permutation tests with 1,000,000 permutations to determine the presence of differences in handedness for each parameter and level of the Maze Navigation Task [43]. To determine between-group differences for each kinematic measure and differences in demographics, we used non-paired permutation tests with 1,000,000 permutations. Between-group differences for each kinematic measure were examined for each level individually. To perform the paired permutation tests, we took the difference between the paired samples of each participant and took the mean of these differences for our initial test statistic. These differences were then resampled–with replacement–for one million iterations, with each iteration calculating a new test statistic. The new test statistic from each iteration is used to create an approximate distribution of values. We counted several values as or more extreme than the positive and negative instances of our initial test statistic, and divided by the total number of iterations to determine our p-value. For non-paired permutation tests, each set of values used to calculate the initial test statistic is resampled–without replacement–for 1,000,000 iterations, with each iteration calculating a new test statistic. To examine differences in behavior as level complexity changes, each participant’s performance across levels was fit with a linear ordinary least squares model for each kinematic measure. The fit of this model was bootstrapped 1,000,000 times where the y-intercept (β_0_) and slope (β_1_) were recorded at each iteration. We took the average of these bootstrapped distributions to obtain a β_0_ and β_1_ to represent each participant. Non-paired permutation tests (described above) were used to determine between-group differences for β_0_ and β_1_ for each parameter. All tests used an alpha level of 0.05 to determine significance. Kinematic data was collected via the tablet application by recording the contact point between the pen surface of the device and was filtered with a low pass, 10 Hz cutoff Butterworth filter commonly used in upper limb kinematic assessments using both robotics and tabled devices [34, 44]. Additionally, the common language effect size (CLES) was calculated for each comparison using the exact method (i.e. comparing all possible combinations between groups) to determine the magnitude of the difference between two groups [45]. Both Permutation tests and the calculation of the common language effect size offer a robust alternative to traditional parametric tests because they do not rely on specific assumptions about the data, such as the assumption of normality or the assumption of equal variances between samples.

## Results

### Participant characteristics

Our study included twenty individuals with stroke (71.75 ± 9.76 years old) and twenty control participants (63.65 ± 12.28 years old). The individuals with stroke were significantly older than controls (p = 0.03, CLES = 68.38). Most participants in each group were right-handed, (Controls: 17, Individuals with Stroke: 17) with three controls and one individual with stroke who was ambidextrous. Details on demographic information are in **Table 1**.

**Table 1:** Participant demographics.

	Controls (n = 20)	Individuals with Stroke (n = 20)
Age	63.65 ± 12.28	71.75 ± 9.76
Sex	Male: 9	Male: 14
	Female:11	Female: 6
Dominant Hand		Left: 2
	Right: 17	Right: 17
	Ambidextrous: 3	Ambidextrous: 1
Months Post Stroke	--	64 [11, 199]
FIM	--	124 [105, 126]
FMA—More Affected	--	63 [11,66]
Field Cut	--	3
Hemisphere of Stroke	--	Left: 9, Right: 11
MOCA	--	24.95 (3.27)
BIT	--	142.5 ± 4.04
PPB—More Affected	--	6.75 [0, 11]
TLT [0,1,2,3] More Affected	--	[18, 1, 1, 0]

Abbreviations: FIM, Functional Independence Measure; FMA, Fugl-Meyer Assessment; MOCA, Montreal Cognitive Assessment; BIT, Behavioral Inattention Test; PPB, Purdue Pegboard; TLT, Thumb Localizing Test. Values are reported as mean ± standard deviation or as Median [Min, Max] Full clinical information can be found in S1 File.

### Differences in temporal and spatial measures

We first examined how performance differed between the dominant and non-dominant hands of controls. For the controls, we found no significant differences in handedness for all levels in Error Time (all levels, p > 0.05), Average Speed (all levels, p > 0.05), Movement Time (all levels, p > 0.05), and Number of Speed Peaks (all levels, p > 0.05). For the Peak Speed measure, we observed a significant difference for one level (Level 7, p = 0.04, CLES = 61.63).

Since we observed a single minor difference between dominant and non-dominant hand performance in controls, we collapsed across handedness for all subsequent analyses. All trial data collected for both hands in controls and individuals with stroke can be found as data in the S1 File.

**Fig 3:**
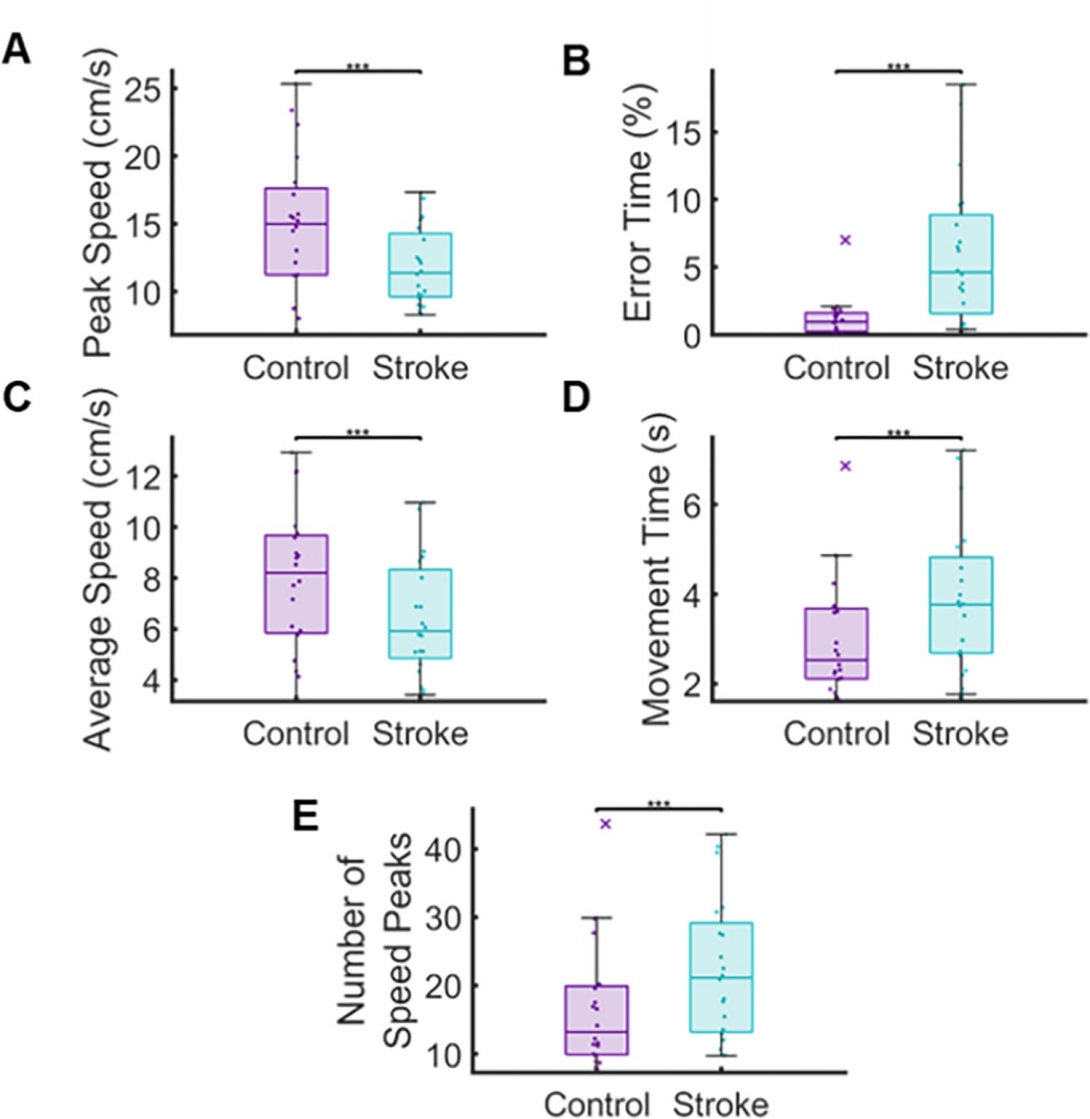
We compared the overall performance Peak Speed (A), Error Time (B), Average Speed (C), Movement Time (D) and Number of Speed Peaks (E) across eight levels. We found significant differences between groups for each measure, indicating that individuals with chronic stroke moved slower, spent more time in contact with the maze boundaries, made more corrective movements, and took more time to navigate the workspace as controls.

To examine the impact of chronic stroke on performance within our temporal and spatial measures, we first compared the overall performance between groups across all levels. For these comparisons, we used the more affected limb of the individuals with stroke **(Fig 3)**. We observed significant differences in overall performance between controls and individuals with stroke in Peak Speed (p < 0.001, CLES = 65.36), Error Time (p < 0.001, CLES = 75.76), Average Speed (p < 0.001, CLES = 66.22), Movement Time (p < 0.001, CLES = 67.05), and Number of Speed Peaks (p < 0.001, CLES = 66.35) (See **Table 2** for full results).

**Table 2:** Parameter values.

	Level Comparison Between Groups								Relationship Across Levels	
Parameters	Levels	Median [Lower Quartile, Upper Quartile]	p			CLES			Median [Lower Quartile, Upper Quartile]	
Peak Speed	Level 1	Control: 18.82 [11.32, 26.40]	0.124			62.00			**β**_**0**_ * Control: 21.82 [13.25, 25.62]	
		Stroke: 14.83 [12.26, 20.36]								
	Level 2	Control: 17.80 [12.12, 22.54]	0.034			69.87				
		Stroke: 13.42 [10.68, 16.44]								
	Level 3	Control: 16.64 [11.09, 21.56]	0.045			73.50			Stroke: 14.37 [11.12, 20.16]	
		Stroke: 13.25 [9.39, 16.08]								
	Level 4	Control: 16.15 [11.39, 20.47]	0.073			67.13				
		Stroke: 12.28 [10.00, 15.65]								
	Level 5	Control: 13.40 [10.94, 16.97]	0.049			71.75			**β**_**1**_ Control: -1.21 [-1.98, -0.52]	
		Stroke: 10.79 [8.30, 13.24]								
	Level 6	Control: 11.15 [8.53, 13.57]	0.002			72.75				
		Stroke: 8.63 [7.23, 10.65]								
	Level 7	Control: 10.68 [8.30, 12.89]	0.185			64.88			Stroke: -0.95 [-1.39, -0.43]	
		Stroke: 8.85 [7.62, 10.98]								
	Level 8	Control: 10.05 [8.14, 12.59]	0.055			67.25				
		Stroke: 8.14 [6.91, 10.62]								
	**Level Comparison Between Groups**								**Relationship Across Levels**	
Parameters	Levels	Median [Lower Quartile, Upper Quartile]	p			CLES			Median [Lower Quartile, Upper Quartile]	
Error Time	Level 1	Control: 0.00 [0.00, 0.95]	<0.001			81.12			**β**_0 ***_ Control: 0.23 [0.00, 1.95]	
		Stroke: 2.44 [0.77, 10.47]								
	Level 2	Control: 0.00 [0.00, 1.56]	0.001			75.46				
		Stroke: 3.24 [0.48, 10.22]								
	Level 3	Control: 0.13 [0.00, 3.04]	0.020			68.68			Stroke: 1.36 [0.76, 10.17]	
		Stroke: 2.90 [0.23, 8.23]								
	Level 4	Control: 0.00 [0.00, 1.48]	< 0.001			72.75				
		Stroke: 1.98 [0.62, 5.56]								
	Level 5	Control: 0.00 [0.00, 1.26]	< 0.001			77/5			**β**_1 ***_ Control: 0.00 [-0.10, 0.13]	
		Stroke: 2.10 [0.43, 7.44]								
	Level 6	Control: 1.38 [0.00, 2.47]	< 0.001			78.81				
		Stroke: 3.52 [0.61, 12.93]								
	Level 7	Control: 0.08 [0.00, 1.09]	< 0.001			75.75			Stroke: 0.07 [-0.07, 0.46]	
		Stroke: 2.62 [0.21, 8.81]								
	Level 8	Control: 0.74 [0.00, 2.67]	0.002			74.63				
		Stroke: 2.64 [0.58, 9.05]								
	**Level Comparison Between Groups**									**Relationship Across Levels**
Parameters	Levels	Median [Lower Quartile, Upper Quartile]		p			CLES			Median [Lower Quartile, Upper Quartile]
Average Speed	Level 1	Control: 11.67 [7.12, 14.57]		0.58			66.88			**β**_0 *_ Control: 13.00 [8.15, 14.08]
		Stroke: 8.45 [7.12, 12.09]								
	Level 2	Control: 10.42 [6.34, 12.30]		0.019			68.88			
		Stroke: 7.68 [5.74, 9.87]								
	Level 3	Control: 9.63 [6.10, 11.10]		0.047			71.88			Stroke: 8.69 [5.51, 11.67]
		Stroke: 7.19 [5.15, 9.25]								
	Level 4	Control: 8.27 [6.02, 10.18]		0.211			69.00			
		Stroke: 6.54 [4.58, 8.18]								
	Level 5	Control: 7.47 [5.78, 9.00]		0.072			67.50			**β**_**1**_ Control: -0.82 [-1.08, -0.52]
		Stroke: 6.04 [4.17, 7.77]								
	Level 6	Control: 5.65 [4.24, 7.42]		0.050			68.88			
		Stroke: 4.40 [3.38, 6.15]								
	Level 7	Control: 5.41 [3.76, 7.28]		0.013			71.13			Stroke: -0.66 [-0.91, -0.38]
		Stroke: 4.16 [3.26, 5.67]								
	Level 8	Control: 5.11 [3.68, 6.47]		0.045			70.00			
		Stroke: 3.78 [2.92, 5.21]								
	**Level Comparison Between Groups**									**Relationship Across Levels**
Parameters	Levels	Median [Lower Quartile, Upper Quartile]		p			CLES			Median [Lower Quartile, Upper Quartile]
Movement Time	Level 1	Control: 1.85 [1.53, 3.07]		0.059			67.63			**β**_**0**_ * Control: 1.58 [1.41, 2.51]
		Stroke: 2.63 [1.87, 3.26]								
	Level 2	Control: 2.05 [1.63, 3.34]		0.025			68.38			
		Stroke: 2.86 [2.13, 3.84]								
	Level 3	Control: 2.03 [1.76, 3.22]		0.041			70.13			
										Stroke: 2.30 [1.66, 4.25]
		Stroke: 2.85 [2.11, 3.89]								
	Level 4	Control: 2.45 [2.00, 3.48]		0.286			68.88			
		Stroke: 3.20 [2.42, 4.52]								
	Level 5	Control: 2.65 [2.11, 3.46]		0.051			65.75			**β**_**1**_ Control: 0.18 [0.15, 0.26]
		Stroke: 3.33 [2.48, 4.85]								
	Level 6	Control: 3.05 [2.21, 4.78]		0.027			67.63			
		Stroke: 3.82 [2.80, 5.46]								
	Level 7	Control: 3.12 [2.31, 4.78]		<0.001			69.88			Stroke: 0.23 [0.17, 0.36]
		Stroke: 4.19 [3.05, 5.38]								
	Level 8	Control: 3.33 [2.55, 4.93]		0.071			69.13			
		Stroke: 4.57 [3.38, 6.00]								
	**Level Comparison Between Groups**									**Relationship Across Levels**
Parameters	Levels	Median [Lower Quartile, Upper Quartile]			p			CLES		Median [Lower Quartile, Upper Quartile]
Number of Speed Peaks	Level 1	Control: 9.5 [6.35, 20.15]			0.048			66.44		**β**_**0**_ Control: 7.35 [4.18, 17.02]
		Stroke: 15.1 [9.05, 20.78]								
	Level 2	Control: 9.00 [6.85, 20.80]			0.024			69.69		
		Stroke: 15.4 [10.05, 22.75]								
	Level 3	Control: 9.5 [6.02, 18.55]			0.042			70.13		
										Stroke: 14.37 [11.10, 20.29]
		Stroke: 14.4 [9.2, 25.45]								
	Level 4	Control: 12.80 [8.05, 20.00]			0.069			67.63		
		Stroke:18.3 [10.85, 30.15]								
	Level 5	Control: 12.6 [9.45, 21.65]			0.082			67.38		**β**_**1**_ Control: 1.38 [0.77, 1.72]
		Stroke: 17.30 [11.8, 30.9]								
	Level 6	Control: 17.80 [12.6, 26.4]			0.020			66.69		
		Stroke: 22.7 [14.6, 32.85]								
	Level 7	Control: 19.20 [13.00, 28.2]			0.011			68.13		
										Stroke: -0.95 [-1.39, -0.41]
		Stroke: 24.2 [15.5, 18.5]								
	Level 8	Control: 18.75 [14.00, 29.25]			0.011			69.19		
		Stroke: 28.5 [18.5, 34.69]								

Median [Lower quartile, Upper quartile] values for Peak Speed, Error Time, Average Speed, Movement Time, and Number of Speed Peaks for both controls and individuals with stroke. Values for individual levels are represented on the left side of the table while bootstrapped coefficients are represented on the right.

**Fig 4:**
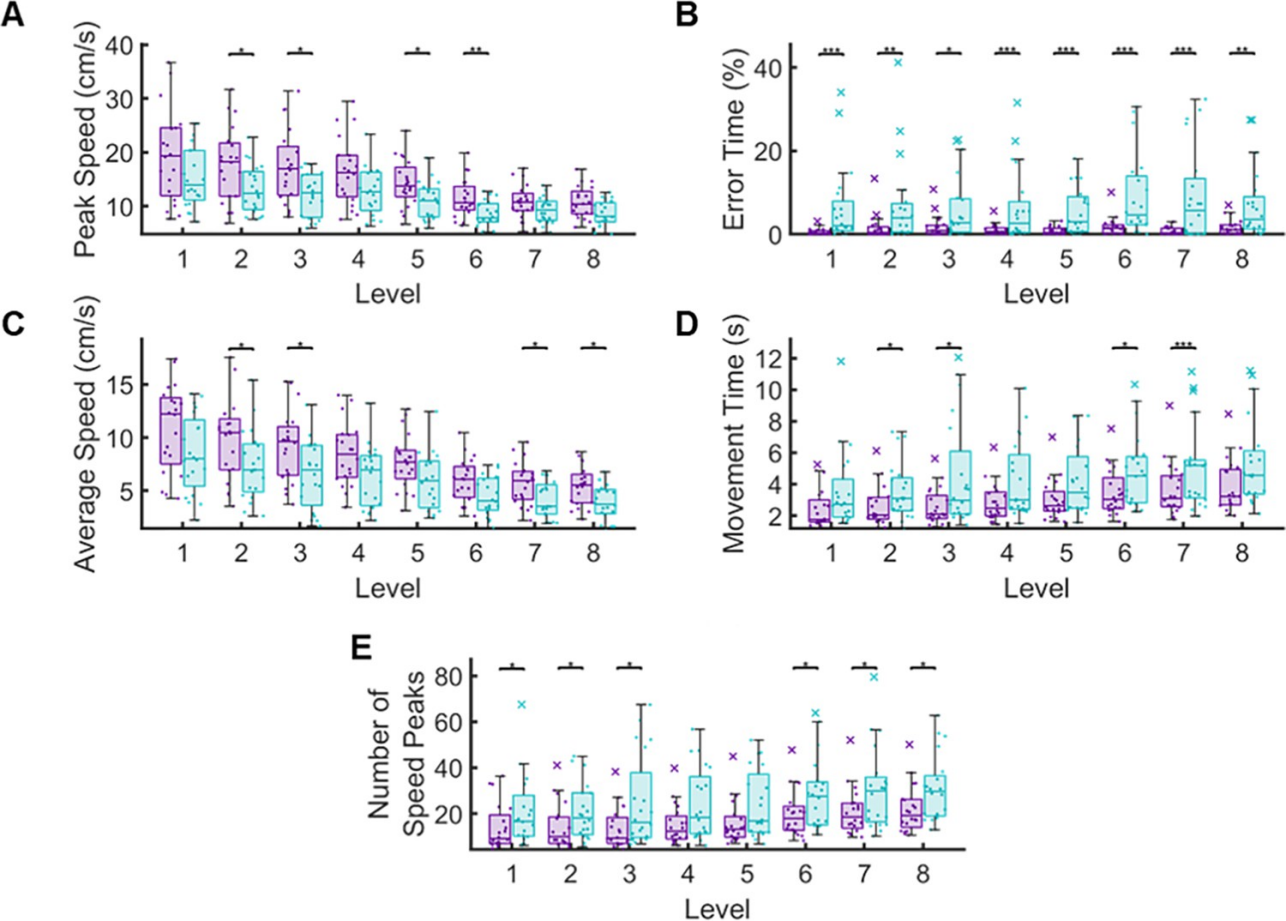
Temporal and spatial accuracy measures for performance across the Maze Navigation Task levels 1–8. We found that individuals with stroke had significantly reduced Average Speed (C) and Peak Speed (A) while having increased Movement Time (D) and Number of Speed Peaks (E) when compared to controls at several levels. While the median Error Time (B) for each level was relatively low for both groups; the individuals with stroke spent significantly more time against or outside the walls of the maze than the controls. (*p<0.05, **p<0.01, ***p<0.001).

To further examine the impact of chronic stroke on required movement complexity, we performed between-group comparisons at each level (**Fig 4).** Unexpectedly, Error Time was the only measure we observed significant differences between groups at every level (all levels, p < 0.001), indicating that individuals with stroke made more errors and spent significantly more time in contact with the boundaries of the maze compared to controls, regardless of maze difficulty (i.e., level). Interestingly for Peak Speed, Average Speed, Movement Time and Number of Speed Peaks we observed significant differences for group behavior in several of eight levels; however, the levels where differences were observed varied between measures. (Peak Speed (Level 2, p = 0.035, CLES = 69.88; Level 3, p = 0.047, CLES = 72.50), Average Speed (Level 2, p = 0.001, CLES = 68.38; Level 3, p = 0.021, CLES = 71.88), Movement Time (Level 2, p = 0.025, CLES = 68.38; Level 3, p = 0.041, CLES = 70.13), and Number of Speed Peaks (Level 2, p = 0.024, CLES = 69.69, Level 3, p = 0.042, CLES = 70.13).We also observed differences in Peak Speed for levels five and six (Level 5, p = 0.049, CLES = 71.38; Level 6, p = 0.003, CLES = 72.75), differences in Average Speed for levels seven and eight (Level 7, p = 0.013, CLES = 71.13; Level 8, p = 0.045, CLES = 70.00), differences in Movement Time for levels six and seven (Level 6, p = 0.027, CLES = 67.63; Level 7, p < 0.001, CLES = 69.88) and differences in Number of Speed Peaks for levels one, six, seven, and eight (Level 1, p = 0.048, CLES = 66.44, Level 6, p = 0.020, CLES = 66.69, Level 7, p = 0.011, CLES = 68.13, Level 8, p = 0.011, CLES = 69.19,) (See **Table 2** for full results). Overall, our results indicate that individuals with chronic stroke are moving slower and taking more time to complete the same mazes as controls. Additionally, we re-ran our analysis excluding 10 individuals with stroke who had MoCA scores less than 26 to identify if cognitive status impacted performance. Within this secondary analysis, we found that the group differences observed between the controls and individuals with stroke were consistent with our original analysis, with significant differences in the overall performance between controls and individuals with stroke for Peak Speed (Control: 14.88 ± 5.15 cm/s, Stroke: 11.21 ± 2.82 cm/s; p < 0.001, CLES = 71.29), Error Time (Control: 1.25 ± 1.52 s, Stroke: 6.84 ± 6.49 s; p < 0.001, CLES = 79.89), Average Speed (Control: 7.94 ± 2.79 cm/s, Stroke: 5.80 ± 2.37 cm/s; p < 0.001, CLES = 74.07), Movement Time (Control: 3.03 ± 1.36 s, Stroke: 4.47 ± 1.89 s; p < 0.001, CLES = 74.86), and Number of Speed Peaks (Control: 16.76 ± 9.47, Stroke: 26.23 ± 11.83; p < 0.001, CLES = 52.22).

### Changes in behavior across levels

**Fig 5:**
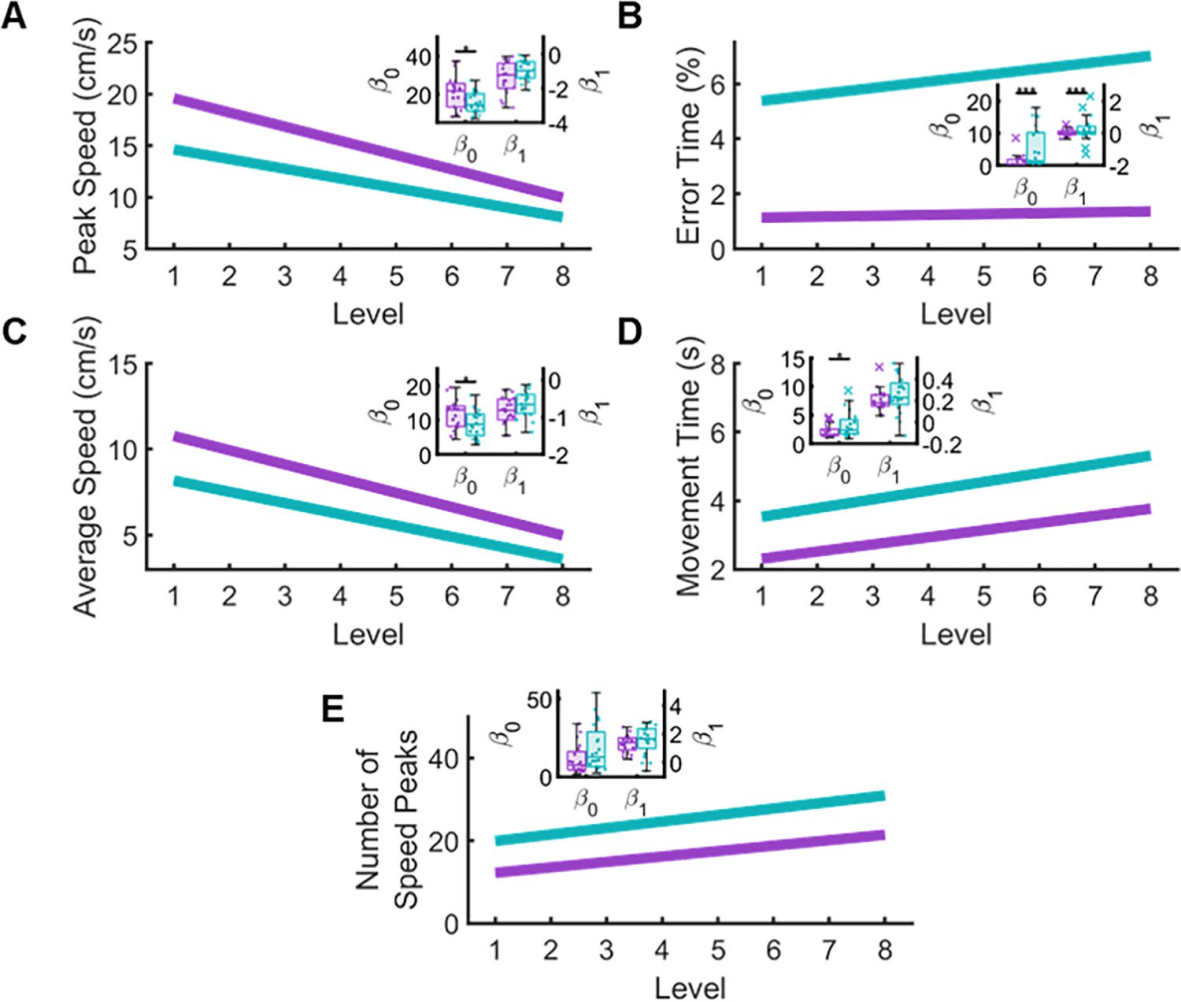
Ordinary Least Squares (OLS) was used to determine the relationship between groups as the level of task complexity increased. β_0_ (y intercept) and β_1_ (slope) were obtained by taking the average of bootstrapped distributions of β_0_’s and β_1_’s after 1,000,000 permutations. Insets in each panel show comparisons between groups of each participants averaged β_0_ and β_1_. Permutation tests between group distributions of β_0_ and β_1_ were used to determine differences between groups. We found that individuals with stroke were significantly impaired in all parameters as indicated by higher β_0_ values. Furthermore, we found that sensorimotor performance diminished as level complexity increased for both individuals with stroke and controls for Peak Speed (A), Average Speed (C), and Movement Time (D) as indicated by similar β_1_ values. Interestingly, we found that as task difficulty increased, Error Time (B) only increased for the stroke group, where Error Time increased as a function of level for individuals with stroke, but remained flat for control participants.

To examine how each group’s performance changed as the number of turns in the maze increased, we compared overall error (β_0_) and the rate of change (β_1_) in performance for each outcome measure between groups **(Fig 5).** To accomplish this comparison, we used ordinary least squares to obtain y-intercepts (β_0_) and a range of slopes (β_1_) that best represent each participants’ performance. Upon comparing β_0_ between groups, we found significant differences in Peak Speed (p = 0.028, CLES = 68.25), Error Time (p < 0.001, CLES = 74.25), Average Speed (p = 0.042, CLES = 69.00), and Movement Time (p = 0.048, CLES = 66.75). This confirms prior analyses calculating an overall error between control and stroke participants (**Fig 3**). In contrast, we observed a significant difference in the rate of change between groups for Error Time (p < 0.001, CLES = 66.5). This indicates that the individuals with stroke increased their Error Time as level complexity increased, while Error Time for controls remained relatively unchanged regardless of task difficulty. No significant differences were observed between groups for β_1_ in Peak Speed (p > 0.05, CLES = 63.00), Average Speed (p > 0.05, CLES = 65.00), and Movement Time (p > 0.05, CLES = 62.00). Furthermore, we observed no significant differences in β_0_ (p > 0.05, CLES = 67.5) and β_1_ (p p > 0.05, CLES = 62.75) for Number of Speed Peaks (Table 2).

### Clinical motor function as a driver for observed sensorimotor differences

**Fig 6:**
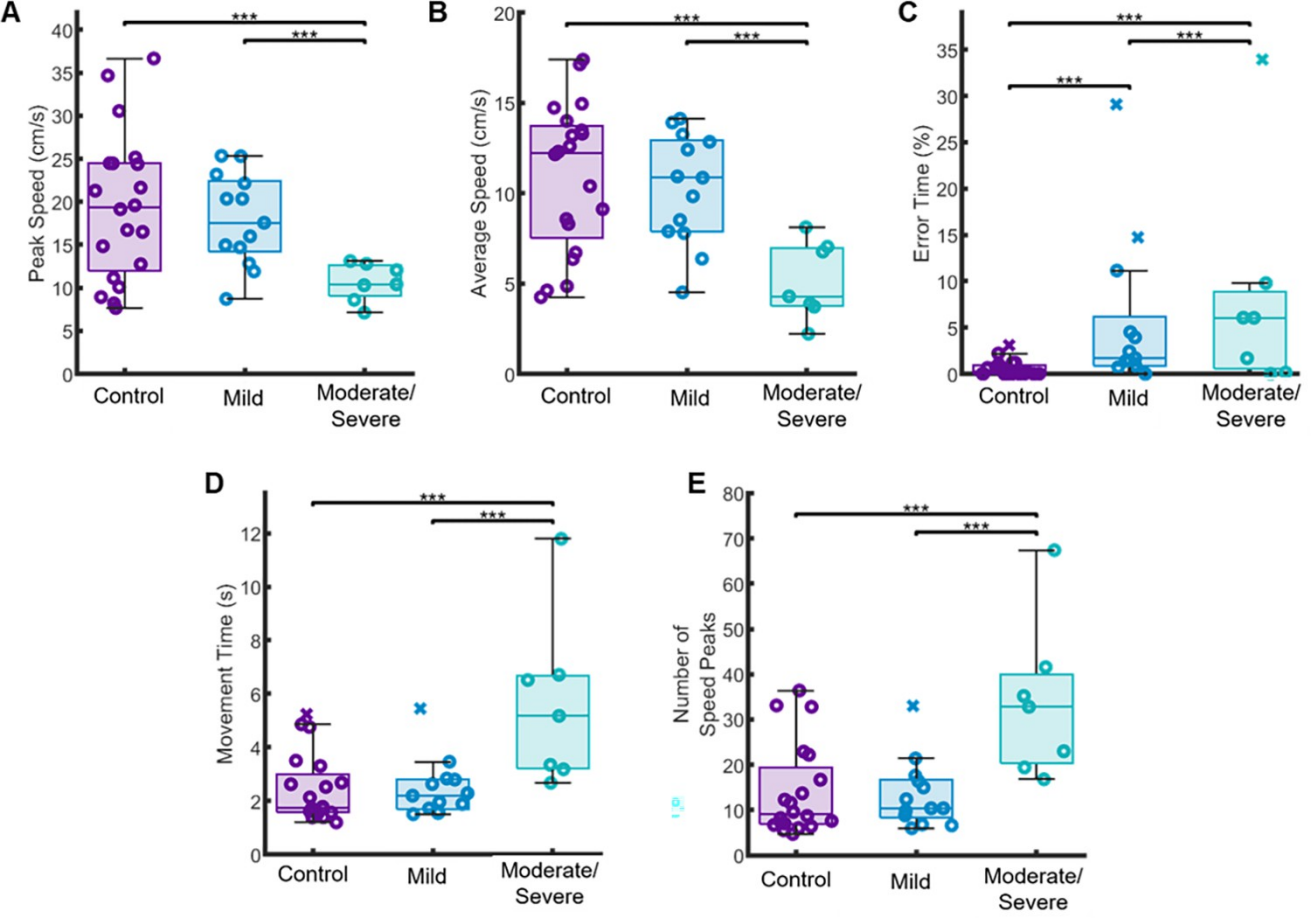
We compared the Fugl-Meyer (FMA) clinical assessment scores for individuals with stroke with their performance for each outcome measure. FMA scores range from 0 (severe hemiparesis) to 66 (normal motor performance). We divided our individuals with stroke into subgroups based on a previously conducted cluster analysis that defined FMA > 53 as mild impairments (N = 13) and FMA < 53 as moderate to severe impairment (N = 7). We found that individuals with stroke with more severe levels of hemiparesis had decreased performance in Peak Speed (A), Average Speed (B), Error Time (C), Movement Time (D), and Number of Speed Peaks (E) when compared to both mild and control groups (***p < 0.001).

Overall, the clinical demographics of our individuals with stroke were broad and ranged from 11–66 on the Fugl-Meyer (Table 1). We completed a secondary analysis to determine the impact of the motor impairments on the Maze Navigation Task by examining the performance of the individuals with stroke after being split into mild (FMA score ≤ 53) and moderate/severe (FMA score > 53) groups **(Fig 6)** [46]. We first compared the performance of our subgroups for each kinematic measure after collapsing across levels. We observed significant differences between subgroups for Peak Speed, Average Speed, Error Time, Movement Time, and Number of Speed Peaks (all p < 0.001, all CLES > 68.83). Additionally, upon comparing the performance of the controls and the mild subgroup of individuals with stroke, we observed no significant differences in Peak Speed, Average Speed, Movement Time, and Number of Speed Peaks (all p > 0.05, all CLES > 64.37). However, we did observe a significant difference in Error Time (p < 0.001, CLES = 89.95).

## Discussion

In this study, we demonstrate the utility of a tablet-based Maze Navigation Task as an effective assessment tool for quantifying complex upper limb reaching movements in individuals with and without stroke. Our findings provide valuable insights into the application of this technique and the unique contributions it offers as an assessment tool. Building upon the foundation of our study’s novel approach, it is evident that accurately quantifying complex upper limb movements presents a considerable challenge, particularly when relying on a limited set of kinematic parameters or when examining individuals with neurological disorders. Moreover, robotic devices with established utility for such quantification are often inaccessible to clinical environments due to high cost [47]. Here, we provide evidence for a tablet-based Maze Navigation Task as an effective assessment tool for quantifying complex upper limb reaching movements after stroke. Additionally, we found that when considering the clinical severity of motor impairment, this task could distinguish between those with stroke who were mildly impaired versus individuals with moderate or severe impairments.

### Utility of tablet-derived kinematics for quantifying impairment

The use of tablets to assess and quantify aspects of rehabilitation and impairment in individuals with neurologic disease and injury has gained popularity in the past decade. This is not surprising given the advances and price reductions in portable technologies, as well as the barriers and time constraints faced by clinicians in neurorehabilitation environments [48]. Recent studies have used a simple finger tapping application (iMotor) on a tablet to quantify hand dexterity in Parkinson’s disease by examining movement timing (finger tapping intervals) and reaction time. This approach effectively distinguished between “ON” and “OFF” medication states in Parkinson’s disease, demonstrating that often very simple kinematics can be used to quantify motor impairment [49]. While this application of kinematics can differentiate between states in Parkinson’s, more complex kinematics and analyses are necessary to survey the wide breath of impairment seen in individuals with stroke. In the field of stroke rehabilitation, the use of tablet or mobile devices has primarily been for administrative purposes during rehabilitation [32, 50–53]. The few studies that have explored the use of tablets to quantify upper limb impairments in individuals with stroke have predominately focused on specific factors of sensorimotor control, such as reaction time or focusing on hand dexterity [29, 54, 55]. Recently, using a variety of kinematics we have demonstrated that reaching behavior in individuals with stroke can be quantified with similar degrees of accuracy to laboratory-based robotic devices [34]. However, a limitation of this work is the simplistic nature of the task, which solely examined simple point-to-point reaching movements in individuals with stroke. In everyday life, the movements we make are much more complex and constantly change to meet the demands of the environment, thus necessitating more tasks that are designed to capture these aspects of movement control, such as our maze navigation task.

Few studies have examined the kinematics of complex limb movements made in individuals with stroke [56–59]. Notably, more complex assessments have been developed for testing upper limb function in individuals with Parkinson’s disease and Multiple Sclerosis using a shape tracing task to effectively predict aspects of clinical function [60]. In the current study, we aimed to develop an objective task that can quantify complex limb movements that closely mirror aspects of “real-world” activity. Our Maze Navigation Task elicits movements akin to those you would make doing a crossword puzzle with a pen or using utensils. The primary goal of this study is to examine critical aspects of motor control in simple environments (a maze with one turn) versus more complex environments (a maze with eight turns)–requiring individuals to frequently adapt their movement strategy to each maze. Surprisingly, only a handful of studies have explored kinematics in realistic tasks or environments that require these kinds of dynamic movements [56, 61–63]. Here, we aimed to create and assess a task that is both user-friendly and portable, all while ensuring the precision of its kinematic measurements.

In this study, we observed that our Maze Navigation Task was capable of distinguishing arm kinematics between controls and individuals with stroke for measures related to movement speed, timing, and error (**Figs 3 and 4**). In three of our five parameters, we found that performance scaled for both controls and individuals with stroke as difficulty of the task increased, with individuals with stroke having significantly higher levels of overall error (**Fig 5**). Notably, the Error Time of controls remained relatively constant regardless of task difficulty. In contrast, the Error Time of the individuals with stroke increased significantly with increases in task difficulty. These results suggest that even within the relatively small workspace of a tablet computer, we can accurately capture impaired motor function and the degradation of this function as environmental constraints become more difficult for the user. Lastly, we did not observe differences between groups for every level in each parameter. The discrepancies between the levels are likely due to how each group handles the varying spatial constraints of the task. For instance, level six has three large channels allowing long-reaching movements, which typically elicit high peak speeds in control participants (Fig 2A). Given that individuals with stroke tend to have slower peak speeds compared to controls for relatively straight reaches of six cm or more, the ample space afforded by the large channels in level six may amplify this distinction [34, 44]. This may indicate that neither a single kinematic or a level is enough to identify the impact of chronic stroke.

### Impact of clinical motor function on performance

To determine if the Maze Navigation Task could effectively distinguish between different levels of motor impairment due to stroke, we separated our individuals with stroke into subgroups using their Fugl-Meyer Assessment (FMA) upper extremity scores [46]. We found that for all measures except Error Time, performance of the mild FMA group (N = 13) was comparable to that of control participants, suggesting that despite having generally “normal” kinematics they still exhibited increased error rates indicative of poor motor control. In contrast, we found that the moderate/severe FMA group (N = 7) was significantly different from the mild FMA group for all measures. This outcome holds significant implications, as it demonstrates that the Maze Navigation Task not only identifies impairments when compared to control participants but also possesses the capacity to discern varying levels of motor impairment among individuals with stroke. Additionally, in three of our five metrics we did not observe significant differences between the controls and mildly impaired individuals with stroke. The UL-FMA assessment used to determine the mild and moderately impaired subgroups only evaluates an individual’s flexion, extension, and range of motion at various joints. Thus, the inability to distinguish between the controls and mildly impaired individuals with stroke likely stems from variations in assessment methodologies between our task and the UL-FMA. Additionally, recent work has demonstrated that the UL-FMA can effectively capture muscle weakness and abnormal muscle synergies in both acute and chronic stages of stroke; however, individuals within the acute phase of stroke often have more impaired reaching kinematics than individuals with chronic stroke with similar UL-FMA scores [64]. This discrepancy suggests that while the UL-FMA effectively quantifies muscle weakness and abnormal synergies, the UL-FMA’s relevance to reaching kinematics may be limited. This observation highlights the potential disparities in assessment methodologies between our task and the UL-FMA, warranting further investigation. The Maze Navigation Task relies on complex interactions between cognitive, motor, and sensory processes to initiate movement, navigate the path, and avoid obstacles (path wall, corners). The initial goal for this task was to examine how changing the complexity of required movements are impacted by stroke. Here, we must note that this task could easily be used to test the influences of cognitive impairment on motor function, as individuals with stroke often have difficulty with spatial perception, planning, and environmental navigation [65–72]. In this study, we screened participants with the MOCA to specifically recruit individuals with no or mild cognitive impairment. Overall, our participants had an average MOCA score of 25, suggesting that these individuals likely did not have concomitant cognitive deficits that would influence task performance. We must note that we tested one participant with a score of 16, which places them in the moderate cognitive impairment range on the MOCA. However, this participant had expressive aphasia and was able to confirm clear comprehension of the task. It is worth noting that administering the MOCA to individuals with aphasia often results in inaccurate scoring due to the MOCA’s strong emphasis on language comprehension [73]. As such, we believe that our current work cannot speak to the influence of cognitive impairment on motor function and future work will aim to determine whether this task can suitably capture cognitive-motor impairments in individuals with stroke.

### Limitations and strategies for the future

One limitation of the current study is an observed difference in age between our control group and our stroke group, with the stroke group being significantly older. While age may have influenced the observed differences between controls and individuals with stroke due to older adults tending to move slower and have more variable movements [74]. We believe the effects of this limitation to be minimal because the average age difference between the controls (63.65 ± 12.28 years old) and the individuals with stroke (71.75 ± 9.76 years old) is only eight years. Upon examination of the kinematic results, the group differences (**Figs 3 and 4**) that are observed are robust and likely would not change with the addition of more participants. To fully characterize the impacts of aging on the execution of complex movement, we aim to survey behavior across decades to determine if there is age-related decline. A second limitation concerns the breadth of parameters included to characterize behavior in this task. The measures included here were based on previous studies examining motor control and task navigation [75]. It is possible that the measures chosen to represent this novel task do not capture the full spectrum of errors made by individuals with stroke. Future work aims to further explore metrics that can capture stability and coordination during task performance such as postural speed and relative time spent navigating corner regions. Not all participants used the assistive devices to complete the task. The three individuals who did use these devices had trouble maintaining an upright grip while holding the pen. This would affect our kinematics since these devices change the wrist orientation needed to complete the task; however, the task was designed to minimize the use of wrist movements due to the size and complexity of each level within the task. When these individuals attempted to perform the task without the devices, it was evident that a lack of wrist and hand stability led to decreases in movement stability and an increase in corrective movements throughout the task. If these individuals performed the task without the assistive devices, it is likely that we would have observed significant increases in the number of errors and/or inability to complete the task. However, these individuals used assistive devices in daily life, so we believe that their performance is indicative of their real-world performance. As we did not asses our individuals with stroke for anxiety or depression, we cannot exclude the impacts of these disorders on motor function such as slowed movement or lack of motivation [76].

## Conclusions

In summary, we created a tablet-based Maze Navigation Task to quantify sensorimotor function in control participants and individuals with chronic stroke. We observed that individuals with stroke were consistently impaired compared to controls–individuals with stroke moved more slowly, took longer to complete the task, and made significantly more errors. Examining performance across levels revealed that individuals with stroke made increasingly more errors when task complexity increased, suggesting that the Maze Navigation Task is suitable for assessing responses to changes in task difficulty or complexity. Lastly, we observed that the task was capable of clearly distinguishing between those with mild vs. moderate/severe impairment of the limb. Our results suggest that the current task has the potential to be a rapid and accurate assessment tool for quantifying sensorimotor behavior after stroke. We believe this assessment is suitable for individuals with a varying degree of motor function and recommend the adaptive devices used within this study if individuals have difficulty maintaining an upright grip on the pen. We would not recommend this assessment to individuals who are unable to maintain an upright sitting posture without assistance to maintain trunk stability.

## Supporting information

S1 File Participant trial data and clinical information for individuals with stroke. (XLSX)
